# Investigation of factors enhancing droplets spreading on leaves with burrs

**DOI:** 10.3389/fpls.2023.1220878

**Published:** 2023-08-17

**Authors:** Pei Wang, Chengrui Xu, Chengsong Li, Lihong Wang, Qi Niu, Hui Li

**Affiliations:** ^1^ College of Engineering and Technology, Key Laboratory of Agricultural Equipment for Hilly and Mountain Areas, Southwest University, Chongqing, China; ^2^ Interdisciplinary Research Center for Agriculture Green Development in Yangtze River Basin, College of Resources and Environment, Southwest University, Chongqing, China; ^3^ National Citrus Engineering Research Center, Chinese Academy of Agricultural Sciences & Southwest University, Chongqing, China

**Keywords:** droplets, high-speed photography, spread area, impact velocity, weed leaves

## Abstract

**Introduction:**

Spread effect is one of the aspects on deposition quality evaluation of pesticide droplets. It could be affected by many factors such as the microstructure of the target plant leaf surface, physical features of the droplets, and the concentration of spray additives.

**Methods:**

In this study, using a high-speed photography system, 2.3% glyphosate ammonium salt solution with different concentration of the additive was applied to investigate the impact process of single droplet deposition on the plant leaf surface with burrs. Effect of droplet sizes and velocities on spreading area and dynamic deposition procedure was analyzed using image processing programs.

**Results:**

The diffusion factor in the process of droplet spreading was changed over time. The occurrence of bubbles in the droplets was observed in the results. With the bubble generation, the droplet diameter expands and a better diffusion effect is obtained. As a result, better spreading effect was obtained as the droplet diameter was expanded with the generation of bubbles. The significant effects of each physical property of droplets on droplet spreading and the interaction effects between the influencing factors were analyzed. A significant correlation was found between additive concentration, droplet impact velocity, droplet diameters and droplet spreading area. All interactions of concentration:velocity, concentration:diameter, velocity:diameter, and concentration:velocity:diameter had a significant effect on the spreading area of droplets. The study of the factors influencing the process of pesticide droplet impact on the leaf surface contributes to the efficient use of pesticides. Thus, the consumption of pesticides and the resulting impact on the environment can be reduced.

## 1 Introduction

Pesticide application is one of the most effective and indispensable means to control pests during crop production, which mainly targets the stems and leaves of the plants (Song et al., 2019). However, environmental pollution and food safety risk issues in crop production could be attributed to the runoff of pesticides to the soil, atmosphere, and water, which could also result in over-residual of agrochemicals in crop products (Xia et al., 2022). Droplet loss to soil can be reduced by increasing the spread area and retention of droplets on plant foliage. The pesticide runoff could be reduced by enhancing droplet spread property to realize a better deposition effect, particularly on leaf surfaces with burrs.

During the deposition procedure, the phenomenon of droplet rebound, splashing, aggregation, and rolling off would occur as the surface of targets might not be effectively wetted (Xu et al., 2021). Droplet retention duration on leaves can affect the absorption of pesticide by insects or plants, leading to different pesticide efficacy (Bukovac et al., 2002; Dong et al., 2015; Krahmer et al., 2021). Microscopic kinetic studies on droplets have shown that the properties of the liquid had a significant influence on the behavior of the droplet when it hits solid objects (Yan et al., 2019). The physical properties of droplets include surface tension, viscosity, density, droplet particle size, impact velocity, and impact angle. Droplets of different surface tensions have different contact angles on crop leaves, thus affecting the adhesion properties of droplets (Kang et al., 2021). Previous studies have shown that the addition of reasonable additives to pesticide formulations could change the physicochemical properties of the solution, thereby improving the wetting deposition of the solution to the target (Appah et al., 2020; Huet et al., 2020; Zhang et al., 2020).

The addition of additives to the spray mixture could enhance the deposition, retention, diffusion, osmosis, and absorption of pesticide ingredients (Xu et al., 2011; Travlos et al., 2017; Li et al., 2019; Lee et al., 2021; Song et al., 2021). On the surface of hydrophilic plants, the droplet fragmentation is related to the leaf surface roughness of plants, while crushing possibility would reduce on the surfaces with polar chemicals of plants such as avocado and cabbage (Massinon et al., 2017).

The spread area and evaporation time of droplets on leaves would also be affected after the addition of additives (Lin et al., 2019). The hydrophilic leaf spread area and evaporation time of two sizes of droplets with and without additives were compared by experimental studies (de Oliveira et al., 2019). It was found that the retention of droplets on the leaf surface is related to the type of leaf surface and the physical and chemical properties of the spray. The above studies presented the effect of additives on droplet spreading and precipitation. However, the mechanism of these changing physical properties was not discussed.

Leaves with burrs are also more water repellent than leaves without trichomes, especially when the trichome density is greater than 1 per 25 mm^2^ (Brewer et al., 1991). The hydrophobicity of the burr surface is related to the villous density, which prevents fluid from reaching the leaf surface, resulting in less retention of the liquid on the leaf surface during application (Xu et al., 2011). It is particularly important to study the impact behavior of pesticide droplets on the burr surface. There were fewer experiments that study the impact of droplets on burr surfaces. In this experimental study, glyphosate and patchouli thistle were used as experimental objects to study the impact behavior of pesticide droplets on the burr leaf surface.

This study investigated the difference in droplet impact behavior on the burr surface under different concentrations of silicone additives. The impact behavior of droplets was analyzed to investigate the effect of pesticide droplet physical properties on droplet spreading on the leaf surface of *Ageratum conyzoides* Linn, a weed species with burr leaves. In turn, optimization strategies for pesticide application parameters could be obtained in order to reduce the environmental pollution by agrochemicals.

## 2 Materials and methods

### 2.1 Test setup

**Figure 1:**
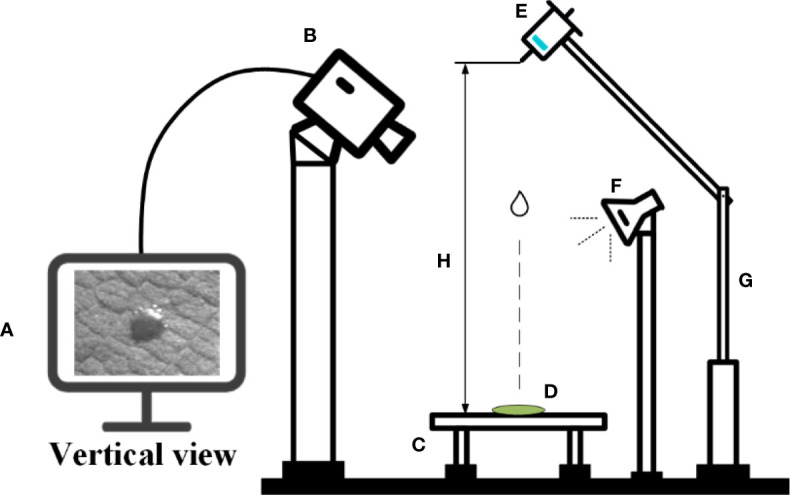
Schematic diagram of the test apparatus. **(A)** Computer + ImageJ software, **(B)** high-speed photography equipment, **(C)** blade holder, **(D)** leaf blade, **(E)** droplet generator pipette, **(F)** LED light source, **(G)** adjustable height holder, and **(H)** droplet release height.

*A. conyzoides* Linn was selected as the pesticide deposition object in this test. It is native to Central and South America and Mexico in North America, and is now widely distributed in tropical and subtropical regions of Asia (Okunade, 2002). During the test, the environmental conditions were controlled at a temperature of 25°C and a relative humidity of 20%. The plant leaves were cut into small blades with a size of 10 × 10 mm. The surface of the blades was wiped with a paper towel and placed on the blade holder. **Figure 1** presents a schematic diagram of the test apparatus. The high-speed photography system (Model: Photron UX50, PHOTRON, Japan) was placed above the blade holder. An LED light source was placed opposite to the high-speed camera. Droplets in volumes of 0.10 μl, 0.15 μl, and 0.20 μl were generated with a pipette gun (Model: 7010101001, DLAB Scientific, Beijing, China, measurement range is 0.1–2.5 μl, minimum adjustable variable is 0.05 μl). The purpose of turning the knob of the transfer gun is to adjust to the proper droplet volume. Then, squeezing the knob of the pipette gun can generate drops of liquid. Droplets were released at heights of 15 cm, 20 cm, and 25 cm. The dynamic impact behavior of droplets hitting the blade and the spreading, bouncing, and balancing of droplets on the blade were recorded by using high-speed photography equipment under a focal length lens of 36 mm. The capture duration was set for 4 s, under a pixel resolution of 1,280 × 512 and a shutter frequency of 4,000 frames per second. The PFV4 (Model: Photron UX50, PHOTRON, Japan) and ImageJ (National Institutes of Health, USA) software were used to view and process the captured images after the shooting was completed.

### 2.2 Chemical preparation

In this study, droplets were generated from 2.3% glyphosate (a.i.: isopropylamine salt, provided by Hebei Zhongbao Green Crop Technology Co., Ltd.) solution mixed with silicone additives (Institute of Plant Protection, Chinese Academy of Agricultural Sciences, 99.9%) in several concentrations of 0%, 0.25%, 0.5%, and 0.75% (**Table 1**).

**Table 1:** Preparation of droplet solutions. V_1_ is the glyphosate volume and V_2_ is the silicone volume.

Concentration	Glyphosate V_1_ (μl)	Silicone V_2_ (μl)	Water (ml)
0.25% silicone	200	25	10
0.50% silicone	175	50	10
0.75% silicone	150	75	10

### 2.3 Physical property evaluation

The physical properties of the droplet itself would greatly affect the spread of the droplet after impacting the blade. The surface tension and contact angle of the droplet would have a greater impact on the spread. Cassie and Baxter (1944) found a relationship between the angle of contact and the solid surface


(1) $$\cos\theta_{c}=f\cos\theta -\left(1-f\right)$$

where f is the solid phase resolution of the solid surface, f <1; $\theta_{c}$ is an apparent contact angle.

**Figure 2:**
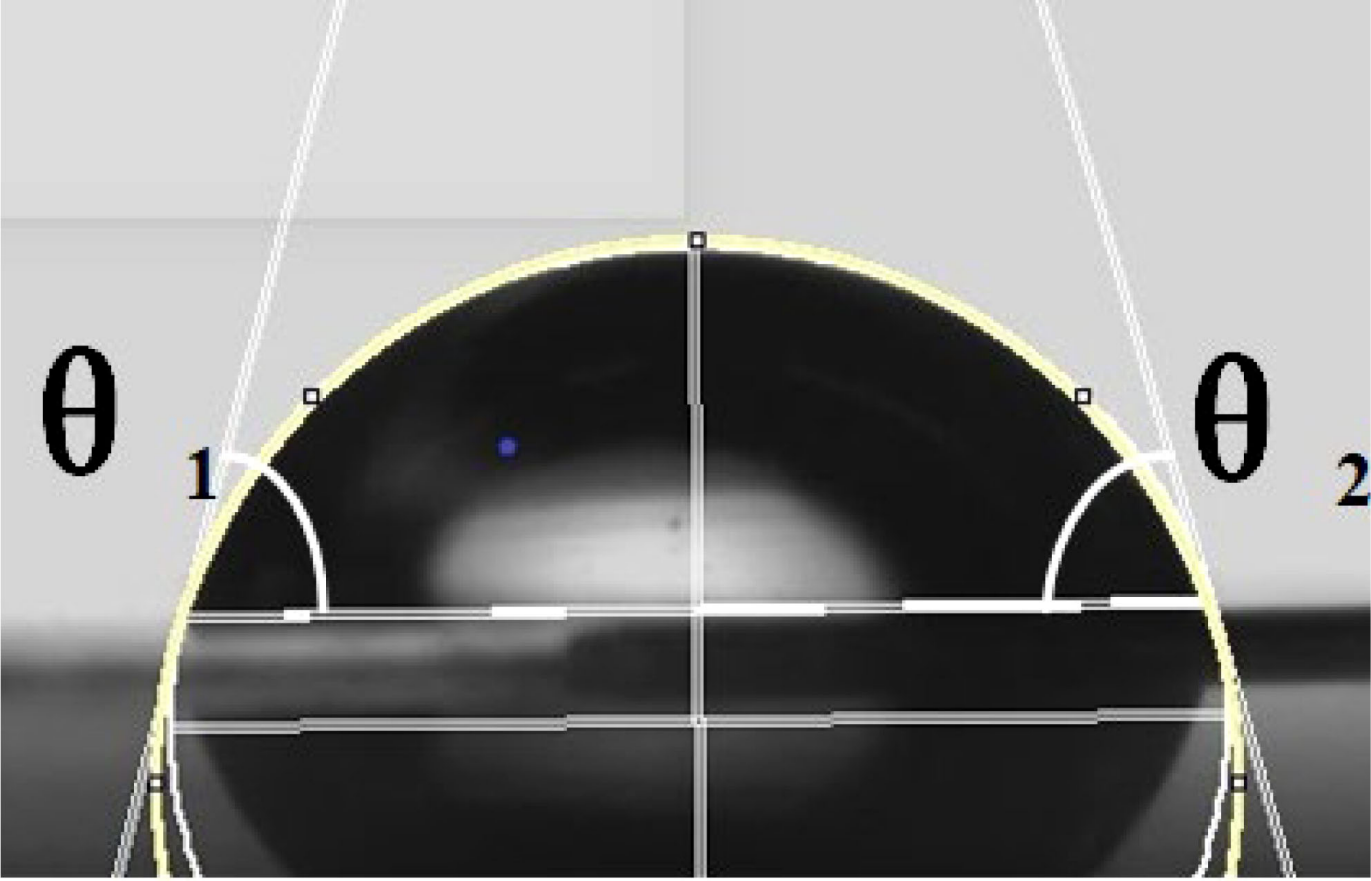
Schematic of contact angle measurement. The yellow line is the contour line of the droplet. The white lines on both sides are the tangent lines of the contact points between the droplet and the surface. θ1 and θ2 are the left and right contact angles, respectively.

It was possible to conclude from the equations that the droplet contact angle would be larger on the solid surface with a lower solid-phase resolution ( f ), which means that solid surfaces with a lower solid-phase resolution would have better hydrophobicity. Thus, the solid surface structure would affect the static contact angle of the droplet, which would further affect the spread and wetting of the droplet after impacting the surface. The lie-down method was applied to measure the static contact angle of the droplet on the solid surface. Leaves of *A. conyzoides* Linn used in the test were fixed on the stage. Eight-microliter droplets were spun out from a micropipette. Small droplets could be delivered to the tested leaf surface by adjusting the micropipette height to ensure that the droplet and plant foliage were just contacted. The measuring system (Kruss, model: DSA10030700, Hamburg, Germany) would correct the droplet shape and measure the contact angle on the leaf surface as shown in **Figure 2**. Each droplet was measured three times in the test and the results are shown in **Table 2**.

**Table 2:** Measurement of static contact angles of glyphosate solutions with different concentrations of additives.

Additive concentration	Number of measurements	Measuring position and measured value (°)			Average (°)	Standard error
		θ_1_	θ_2_	mean		
	1	66.975	68.199	67.587		
0%	2	65.772	66.975	66.373	65.609	2.000772
	3	62.301	63.435	62.868		
	1	61.189	62.301	61.745		
0.25%	2	59.036	59.036	59.036	58.28	3.183449
	3	54.058	54.058	54.058		
	1	44.293	44.293	44.293		
0.50%	2	45.725	46.469	46.097	45.747	1.072882
	3	46.469	47.231	46.85		
	1	38.108	38.66	38.384		
0.75%	2	38.66	39.226	38.943	39.344	1.418781
	3	41.634	41.634	41.634		

**Figure 3:**
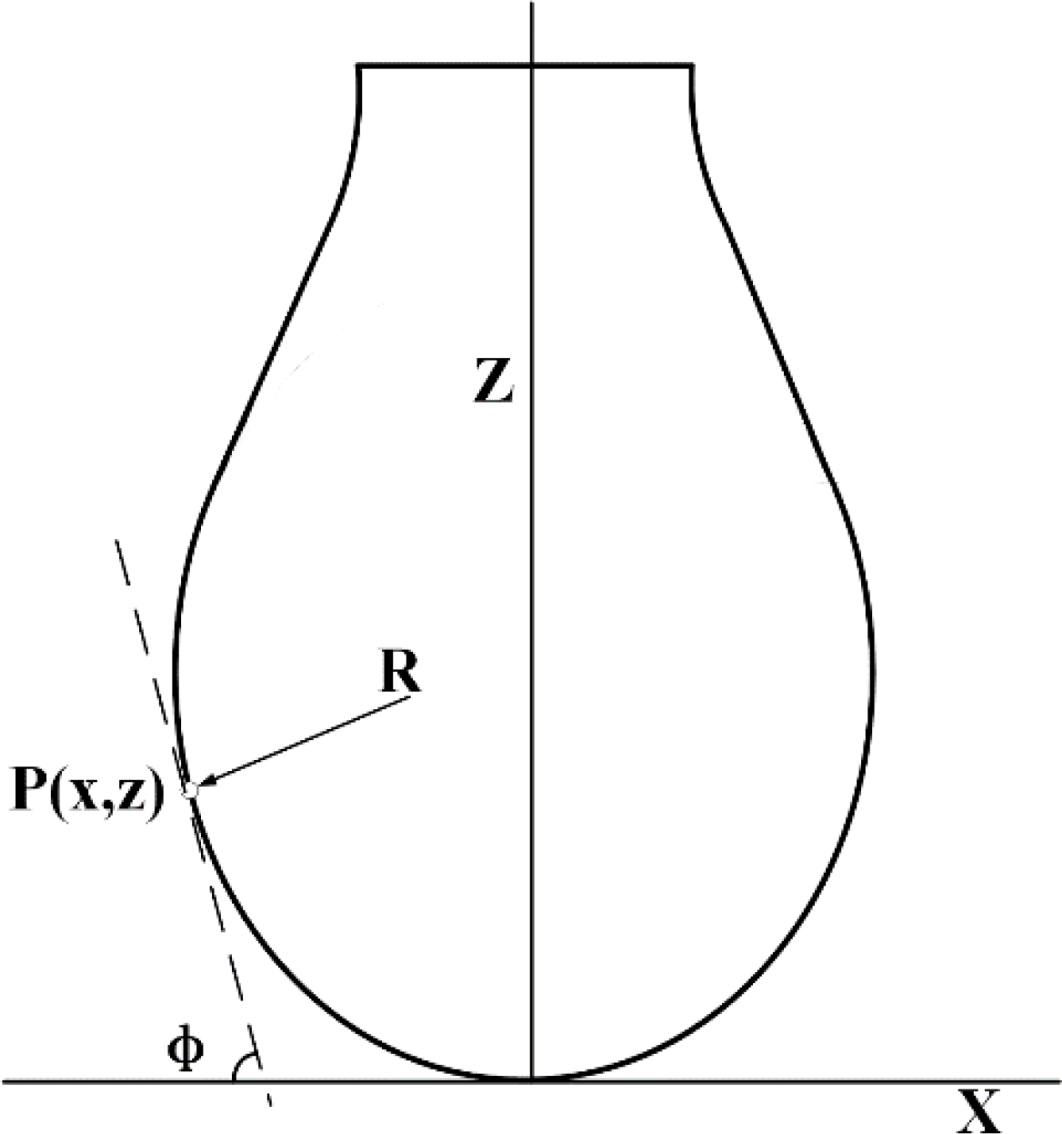
Schematic of surface tension measurement. R is the radius of curvature of a point P (*x*, *y*) on the contour of the drop in the plane; Ф is the angle between the tangent line and the *x* axis at the point P (*x*, *y*) on the contour line; Z and X correspond to the *X* and *Z* axes respectively.


The surface tension of the droplets was measured using the suspension method. The shape of large droplets or bubbles was varied due to the competition between gravity and cohesion between liquid molecules. Gravitational forces tended to lengthen suspended droplets, while cohesive forces tended to produce compact, spherical droplets. The surface tension could be compared using capillary length by analyzing the equilibrium droplet shape (**Figure 3**). If the density of the fluid and the surrounding medium was known, the surface tension could be calculated. The specific principle is

(2) 2−β(z/b)=b/R+bsinФ/x

(3) β=(b^2×Δρ×g)/γ=b2/α2

(4) $$\text{α}=\sqrt{\left(\text{γ}/\left(\Delta \text{ρ}\times \text{g}\right)\right)}$$

where b is the radius of curvature at the low end of the suspension droplet; R is the radius of curvature of a point P(*x*,*y*) on the contour of the drop in the plane; Ф is the angle between the tangent line and the *x* axis at the point P(*x*,*y*) on the contour line; β is the shape factor of the droplet, which determines the shape of the droplets; Δρ is the density difference between the liquid direction and the surrounding gas phase; γ is the surface tension; and α is the capillary constant of the system.

The system software was used for the image processing in this test. The surface tension was measured by matching the suspension drop profile with the program. Surface tension was measured using the contact angle measuring instrument as described above. The surface tension of each droplet was measured three times during the test. The results are shown in **Table 3**.

**Table 3:** Measurement of surface tension of glyphosate solutions with different concentrations of additives.

Additive concentration	Number of measurements	Measured value (mN/m)	Average (mN/m)	Standard error
	1	43.746		
0	2	43.613	43.591	0.136428
	3	43.414		
	1	30.732		
0.25%	2	30.749	30.76	0.02798
	3	30.798		
	1	24.247		
0.50%	2	23.782	23.868	0.280654
	3	23.576		
	1	22.854		
0.75%	2	23.132	23.12	0.212459
	3	23.374		

## 3 Results

### 3.1 Spreading process

**Figure 4:**
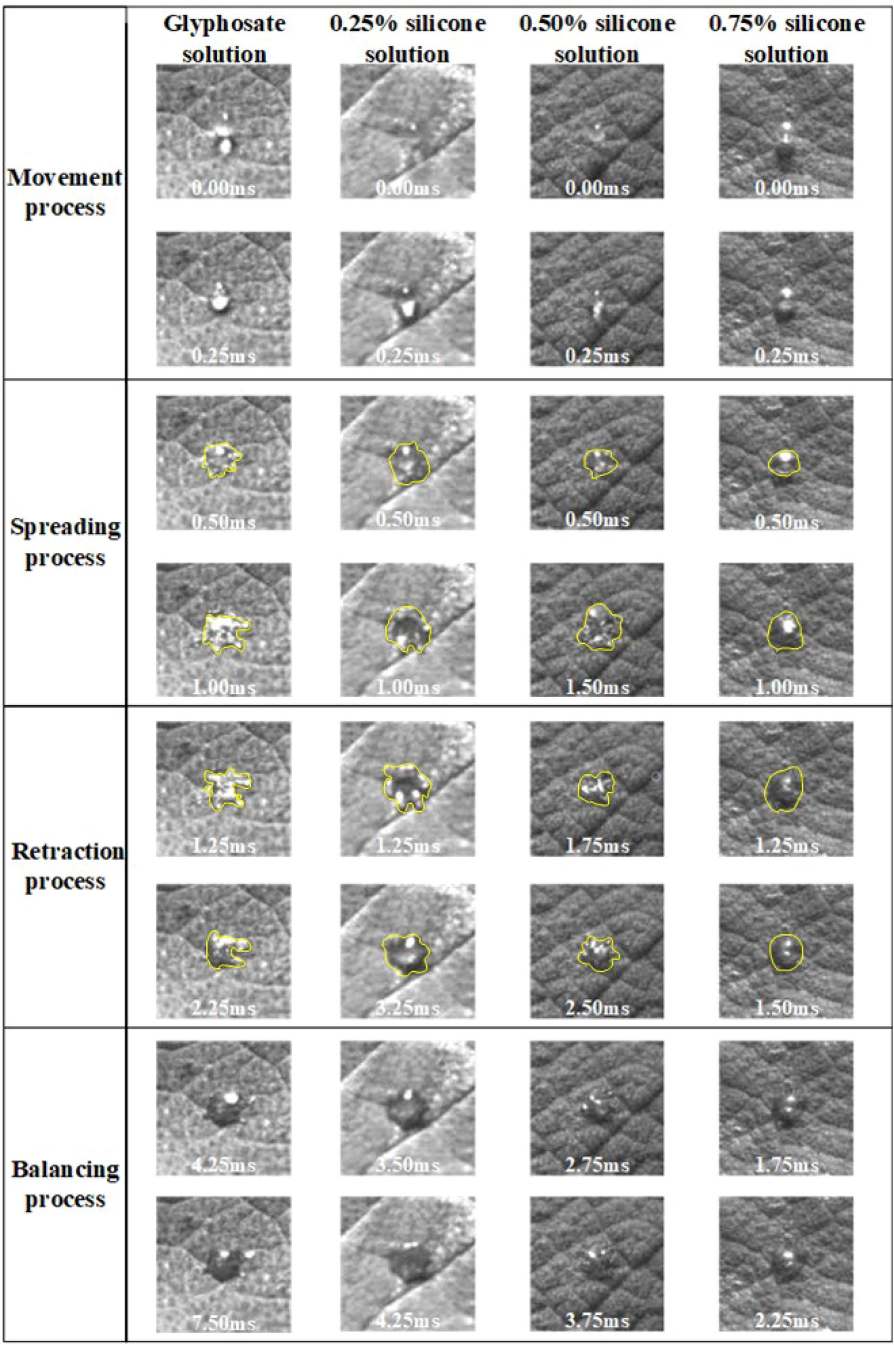
Spreading process of droplets after impacting the blade surface. The spreading process of droplets of 2.3% glyphosate solution with organosilicon additive concentrations of 0%, 0.25%, 0.50%, and 0.75%, respectively, with a velocity of 1.776 m/s and a diameter of 576 μm, impacted the leaf surface. Different categories of images have different scales.

After the droplet affected the burr surface, there were four processes: movement, spreading and fragmentation, retraction, and balancing. No rebound behavior was observed in this test. **Figure 4** shows the impact process on burr leaves of glyphosate droplets with 0.25%, 0.50%, and 0.75% silicone additives released at a height of 15 cm and with a diameter of 576 μm. The duration *t* was set as 0 ms when the droplet reached the leaf blade. The next image was recorded immediately after the contact of the droplet and leaf was defined as the beginning of spreading, when *t* = 0.25 ms (the minimum time interval between two shutters). Since 0.25 ms onwards, the droplets began to spread on the leaf surface. As shown in **Figure 4**, in the initial spread of droplet of glyphosate solution without additives, the edge of the deposition area was disturbed by the fine villi on the leaf surface. The surface tension was large, resulting in the spreading edge showing divergent spreading. This situation resulted in a splash of tiny droplets on impact. Test results in **Table 3** show that droplet surface tension reduced when the silicone additive was mixed with the glyphosate solution. The low surface tension made the droplets less capable of maintaining the spherical shape.

**Figure 4** shows that, at the beginning of the spreading process, the droplets with adjuvant could spread smoothly despite the disturbance from the burr on the leaf surface. A more regular circle was formed as the edge of the deposited area. Droplets spread to the maximum area approximately t = 1.00 ms. During the droplet spreading, the kinetic energy of the droplets was dissipated to overcome the viscosity and surface tension, which induced the establishment of a new droplet shape. Glyphosate solution droplets were subjected to excessive resistance in spreading due to their surface tension and the surface villi of the leaves, resulting in incomplete spreading. During the spreading process, droplets with additives also spread more widely than the droplet of glyphosate-only solution.

The droplets shown in **Figure 4** started to retract at *t* = 1.25 ms. The droplet retraction was affected by the surface tension of the droplets, the internal intermolecular forces, and the magnitude of the adsorption force on the droplet surface. The surface tension acting on the surface of the droplets prevented the droplets from spreading on the blade surface and thus caused the droplets to retract. It can be seen from **Figure 4** that the retraction time of droplets with different additive concentrations was not the same. The retraction time of glyphosate solution with maximum surface tension was 1 ms, while the retraction time of the droplets with additive concentrations of 0.25%, 0.5%, and 0.75% was 2 ms, 1.25 ms, and 0.25 ms, respectively. It could be seen that the shape of the four droplets differed greatly after retraction. The degree of completion of the retraction was mainly affected by the surface tension and the surface structure of the blades. Droplets with 0.75% silicone had bubbles inside the droplet before impact. It also presented better spreading effect and shorter retraction time of droplets with 0.75% silicone.

### 3.2 Spread area

#### 3.2.1 Relationship between spread area and droplet diameter

**Figure 5:**
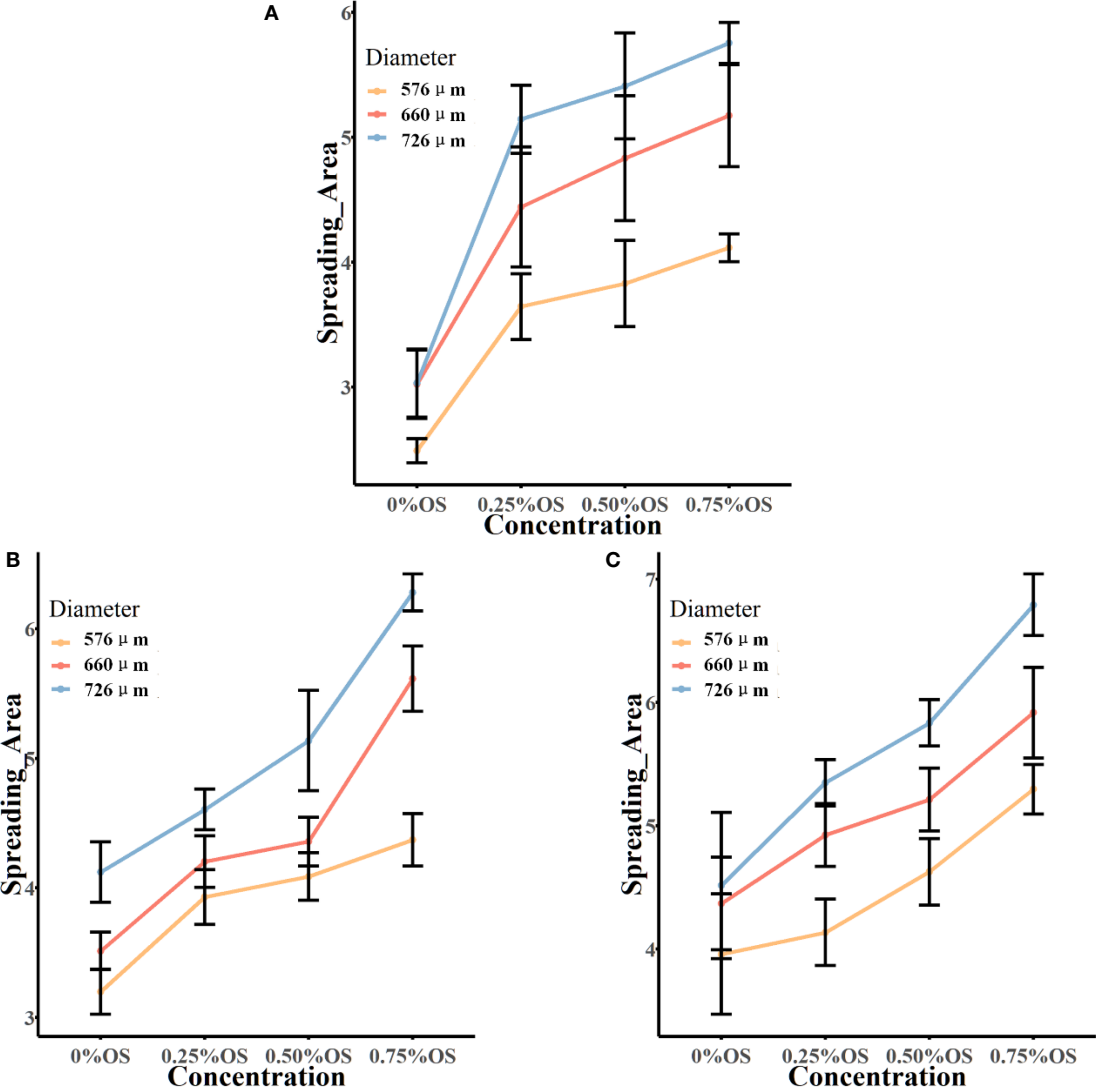
The spread area of the droplet impacting the burr surface. Panels **(A-C)** show the final spread area after impacting the leaf surface at a velocity of 1.776 m/s, 1.979 m/s, and 2.212 m/s, respectively. In the figure, the vertical coordinate is the spread area of droplets on the leaf surface; the horizontal coordinate is the solution of glyphosate with different concentrations of additives. OS, organic silicone. Spread area unit: mm².

**Figure 5** presents the results of the spread area of glyphosate solutions with different additive concentrations, different diameters, and different velocities. **Figure 5A** shows that the spread area of droplets with 0.25% silicone increased by 21.91% when the diameter increased from 576 μm to 660 μm. When the droplet diameter increased to 726 μm, the spread area of droplets increased by 41.17% and 15.81% compared with the spread area of droplets in diameters of 576 μm and 660 μm, respectively.

The spread area of droplets of the silicone additive at 1.776 m/s that affected the blade can be seen in **Figure 5** for an additive concentration increase to 0.50% silicone additive. The spread area of droplets and droplet diameters could be seen to be positively correlated at three velocities. The spread area after the impact of 660-μm diameter at a velocity of 1.776 m/s increased by 26.23% compared with the spread area after the impact of 576 μm. The spread area increased by 41.32% after the droplet diameter increased to 726 μm and the spread area increased by 11.96% after the droplet diameter increased from 660 μm to 726 μm. At the rate of 1.979 m/s and 2.212 m/s, the glyphosate solution of 0.5% silicone additive was added, and the spread area of the droplets increased with the increase in diameter.

#### 3.2.2 Relationship between spread area and additive concentration

As shown in **Figure 5A**, after adding 0.25% silicone additives, the droplets in all the three diameters presented a significant increase of the spread area after impacting the blade compared to the spread area of droplets without the addition of silicone additives. The addition of additives to the glyphosate solution could greatly increase the spread area of the droplets.

At a velocity of 1.776 m/s, the droplet diameter was 576 μm, and the spread area of the solution with 0.25% silicone additives was 46.36% higher than that of the solution without additives, which was a larger increase. With the increase of the concentration of the additives, the spread area of the silicone solution added to 0.5% was only 5.1% higher than that of the 0.25% silicone solution, and the 0.75% silicone solution was increased by 7.5% compared with the 0.5% silicone solution. It could be seen to increase the concentration of the additives, and the spread area of the droplet could also be increased. The same situation was also reflected in the droplets with droplet diameters of 660 μm and 726 μm. In droplets with a diameter of 660 μm, for each increase in silicone concentration of 0.25%, the growth of the spread area was 47%, 8.8%, and 7.0%, respectively. For each increase in silicone concentration of 0.25% of droplets with a diameter of 726 μm, the growth of the spread area was 69.73%, 5.2%, and 6.4%, respectively.

From **Figure 5B**, the spread area of droplets with a diameter of 576 μm increased by 22.87%, 4.1%, and 6.9%, respectively, with the concentration of the additive 0%–0.75% at a velocity of 1.979 m/s. The same situation was also observed for droplets of 660-μm and 726-μm diameters. It can be seen that at a velocity of 1.979 m/s, the spread area of the droplet increased with the increase of the concentration of silicone additives. The spread area of the droplet after impacting the blade at a velocity of 2.212 m/s is shown in **Figure 5C**. At this velocity, it can also be seen that the droplet spread area is increasing with increasing concentration of silicone additives.

#### 3.2.3 The relationship between spread area and velocity

The velocity of droplets impacting the leaf surface was controlled by releasing droplets at different heights in the experiment. Find the local acceleration of gravity and use the equation *v* = √2*gh* to calculate the velocity of the droplet at impact, where *v* is the droplet falling velocity, *g* is the acceleration of local gravity, and *h* is the droplet release height. **Table 4** shows the spread area of droplets at different velocities and the growth of droplet spread area when comparing different velocities. The spread area of glyphosate droplets without additives increased with increasing droplet impact velocity at three diameters. The same situation was observed in glyphosate solutions with 0.75% silicone additives. The impact velocity of 576-μm droplets was increased from 1.776 m/s to 2.212 m/s, and the growth of the spread area was 6.22%, 21.17%, and 28.71%. The droplet spread area of 660 μm and 726 μm was also continuously increased after the velocity increase. However, in glyphosate solutions with 0.25% and 0.50% silicones added, the effect of the increase in velocity on the droplet spread area was not clear.

**Table 4:** Droplet spread area. v1: 1.776 m/s; v2: 1.979 m/s; v3: 2.212 m/s.

Solution	Diameter	Spread area (mm^2^)					
		Velocity			Percentage increase		
		v1	v2	v3	v2-v1	v3-v2	v3-v1
2.3% glyphosate	576 μm	2.489	3.197	3.958	28.45%	23.80%	59.02%
	660 μm	3.021	3.513	4.369	16.29%	24.37%	44.62%
	726 μm	3.030	4.121	4.514	36.01%	9.54%	48.98%
2.3% glyphosate + 0.25% organic silicone	576 μm	3.643	3.928	4.134	7.82%	5.24%	13.48%
	660 μm	4.441	4.202	4.924	-5.38%	17.18%	10.88%
	726 μm	5.143	4.605	5.348	-10.46%	16.13%	3.99%
2.3% glyphosate + 0.50% organic silicone	576 μm	3.828	4.088	4.626	6.79%	13.16%	20.85%
	660 μm	4.832	4.357	5.211	-9.83%	19.60%	7.84%
	726 μm	5.410	5.136	5.833	-5.06%	13.57%	7.82%
2.3% glyphosate + 0.75% organic silicone	576 μm	4.114	4.370	5.295	6.22%	21.17%	28.71%
	660 μm	5.172	5.615	5.916	8.57%	5.36%	14.39%
	726 μm	5.754	6.279	6.790	9.12%	8.14%	18.00%

#### 3.2.4 Interpretation of the ANOVA results

##### 3.2.4.1 Main effects

The ANOVA results presented sufficient statistical evidence that the concentration, velocity, and diameter could significantly affect the spread area of the droplets, as shown in **Table 5**. The *post-hoc* analyses showed that 0.75% of the concentration had the highest effect with a mean of 5.086587, while 2.212 m/s of velocity had the highest effect with a mean of 4.863929.

**Table 5:** Analysis of variance table.

	Df	Sum	Mean_sq		*F*-value	Pr(>*F*)
Replicationvector	5	6.919	1.3838		6.6433	9.100e-06 ***
Fact.A	3	77.837	25.9458		124.5629	< 2.2e-16 ***
Fact.B	2	20.922	10.4609		50.2215	< 2.2e-16 ***
Fact.C	2	56.094	28.047		134.6507	< 2.2e-16 ***
Fact.A:Fact.B	6	5.853	0.9755		4.6831	0.0001702 ***
Fact.A:Fact.C	6	8.251	1.3752		6.602	2.069e-06 ***
Fact.B:Fact.C	4	2.026	0.5064		2.4312	0.0486874 *
Fact.A:Fact.B:Fact.C	12	12.424	1.0354		4.9706	3.038e-07 ***
Residuals	211	43.95	0.2083			

Replicationvector, repeat groups of measurements; Fact.A, concentration; Fact.B, velocity; Fact.C, diameter. p < 0.05: indicated by an asterisk "*". This indicates that the observed result is statistically significant at the significance level α = 0.05.p < 0.01: indicated by two asterisks "**". This indicates that the observed result is highly significant at the significance level α = 0.01. p < 0.001: ndicated by three asterisks "***". This indicates that the observed result is highly significant at a significance level of α = 0.001.

##### 3.2.4.2 Interaction effect

The two-factor interaction between concentration and velocity reported a *p*-value of 0.0001702. This was less than alpha (0.05). Concluding with 95% statistical confidence, sufficient data existed to evidence that the interaction of concentration and velocity could affect the spread area of droplets. The *post-hoc* LSD test showed that the combination of 0.75% concentration and 2.212 m/s velocity led to the maximum spread area with a mean of 5.364571 mm², while the combination of 0% concentration and 1.776 m/s velocity showed a low effect on the droplet spreading with a mean of 2.953095 mm².

The two-factor interaction between velocity and diameter reported a *p*-value of 0.0486874. This was less than alpha (0.05). Concluding with 95% statistical confidence, it indicated that the combination of velocity and diameter had a significant effect on the spread area. *Post-hoc* analyses showed that 2.212 m/s:726 μm exhibited the highest effect with a mean of 5.245607, while 1.776 m/s:576 μm exhibited the lowest effect with a mean of 3.477071.

The two-factor interaction between concentration and diameter reported a *p*-value of 2.069e-06. This was less than alpha (0.05). Concluding with 95% statistical confidence, it indicated that the combination of concentration and diameter had a significant effect on the spread area. *Post-hoc* analyses showed that 0.75%:726 μm exhibited the highest effect with a mean of 5.783143, while 0%:576 μm exhibited the lowest effect with a mean of 3.217667.

The three-factor interaction of concentration:velocity:diameter reported a *p*-value of 3.038e-07. This was less than alpha (0.05). Concluding with 95% statistical confidence, there existed sufficient statistical evidence to support the claim that the three-factor interaction of concentration, velocity, and diameter had a significant effect across all sets of combinations. *Post-hoc* analyses showed that 0.75%:1.979 m/s:726 μm exhibited the highest effect with a mean of 5.934429, while 0%:1.776 m/s:576 μm exhibited the lowest effect with a mean of 2.488571.

**Figure 6:**
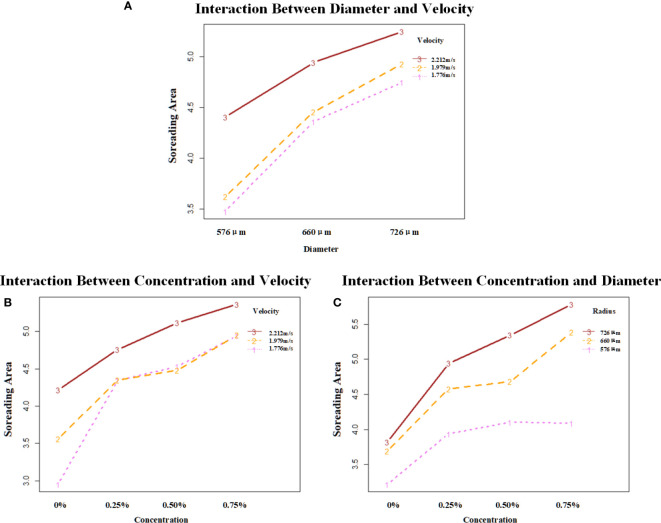
Analysis of the interaction effect between each of the two factors. **(A)** shows the interaction effect between diameter and velocity; **(B)** shows the interaction effect between concentration and velocity; **(C)** shows the interaction effect between concentration and diameter. The vertical coordinates are the mean values of spread area under different combinations of factors. Velocity unit: m/s; diameter unit: μm.

It could be seen from **Figure 6** that the droplet with a velocity of 2.212 m/s has the best spreading effect at each diameter with the interaction of droplet diameter and velocity, in which the spread area of droplets with three velocities at all three droplet diameters has a positive trend. With the interaction of droplet additive concentration and velocity, the droplet with a velocity of 2.212 m/s has the best spreading effect at each additive concentration. Droplets with velocities of 1.979 m/s and 1.776 m/s had similar effects with the addition of additives. The spread area of the droplet at all three droplet velocities at all three additive concentrations showed a positive trend. The droplet with a diameter of 726 μm had the best spreading effect at the three additive concentrations with the interaction of droplet diameter and additive concentration. The droplet with a 576-μm diameter showed no growth trend at 0.5%–0.75% of the additive concentration. Droplets with 726-μm and 576-μm diameters showed a positive growth trend in the spread area at all three additive concentrations.

### 3.3 Spreading factors

**Figure 7:**
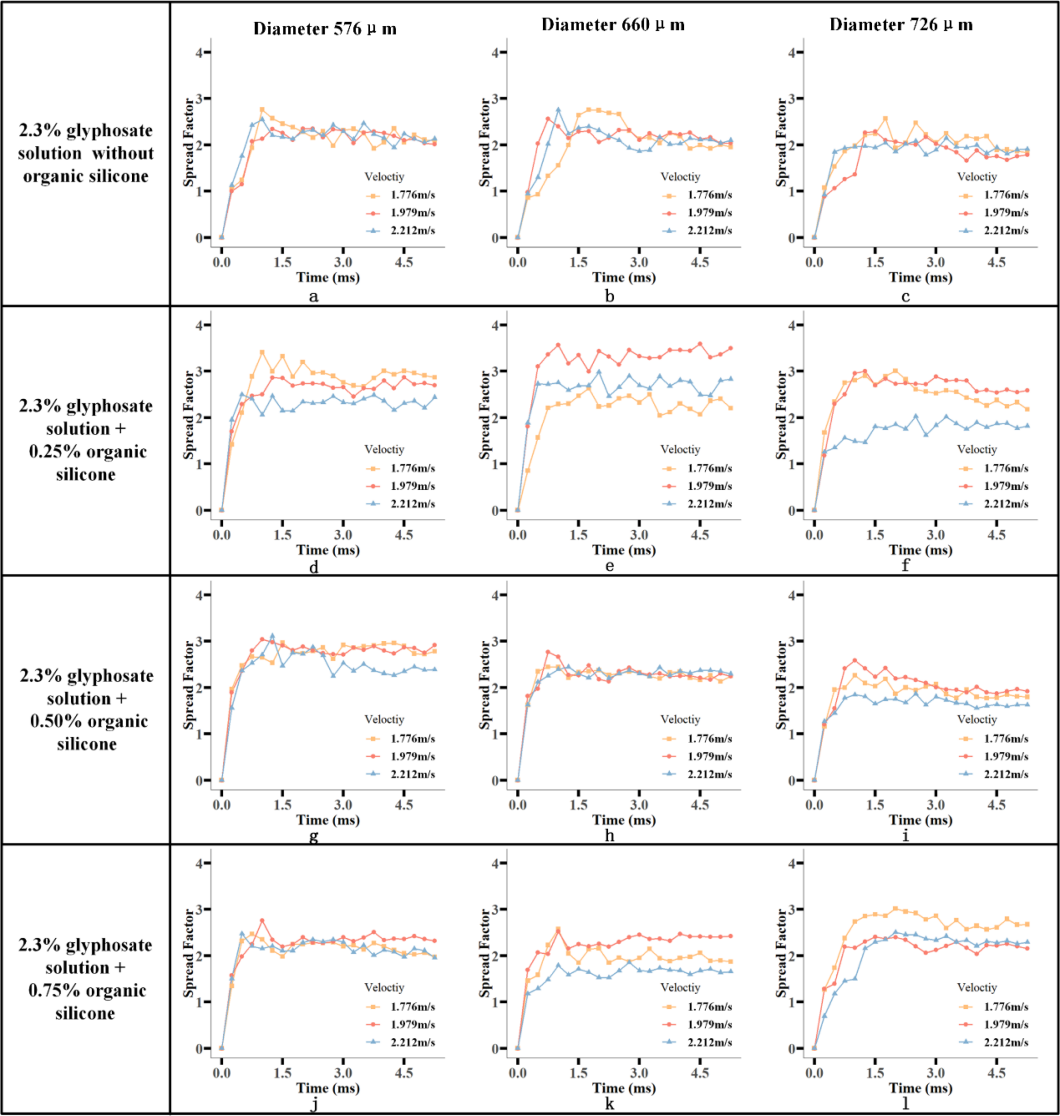
Variation of spreading factor ( $\xi_{t}$ ) after the droplet affected the leaf surface. Evolution of the diffusion factor of droplets impacting the leaf surface over time for droplets of 2.3% glyphosate solution with organosilicon additive concentrations of 0%, 0.25%, 0.50%, and 0.75% at velocities of 1.776 m/s, 1.979 m/s, and 2.212 m/s and diameters of 576 μm, 660 μm, and 726 μm, respectively. The first to third columns are droplet spreading factors with diameters of 576 μm, 660 μm, and 726 μm, respectively. For ease of presentation each image is numbered from **(A-L)**.

To describe the impact phenomenon in more detail and accurately, the time evolution of the spreading factor (defined as spread factor, $\xi_{t}=D_{t}/D_{0}$ , where $D_{0}$ is the initial diameter of the droplet before impact and $D_{t}$ is the spreading diameter of the droplet impacting blade at time t) of the four solutions at different velocities and different diameters is shown in **Figure 7**. **Figures 7A-C** show the spreading process of the glyphosate solution without the addition of silicone additives at 576-μm, 660-μm, and 726-μm droplet diameters impacting the leaf surface, respectively. In **Figure 7A**, it can be seen that for the 576-μm-diameter droplets, the droplets of three velocities reached the maximum spread area at the time of 1.00 ms to 1.25 ms. The degree of undulation of the variation curve of the spreading factor of these droplets is relatively small at the three velocities. There was no significant increase or decrease in the spreading factor. However, the fluctuation of spreading factor variation for droplets with diameters of 660 μm and 726 μm was slightly higher than that for droplets with diameters of 576 μm.

**Figure 8:**
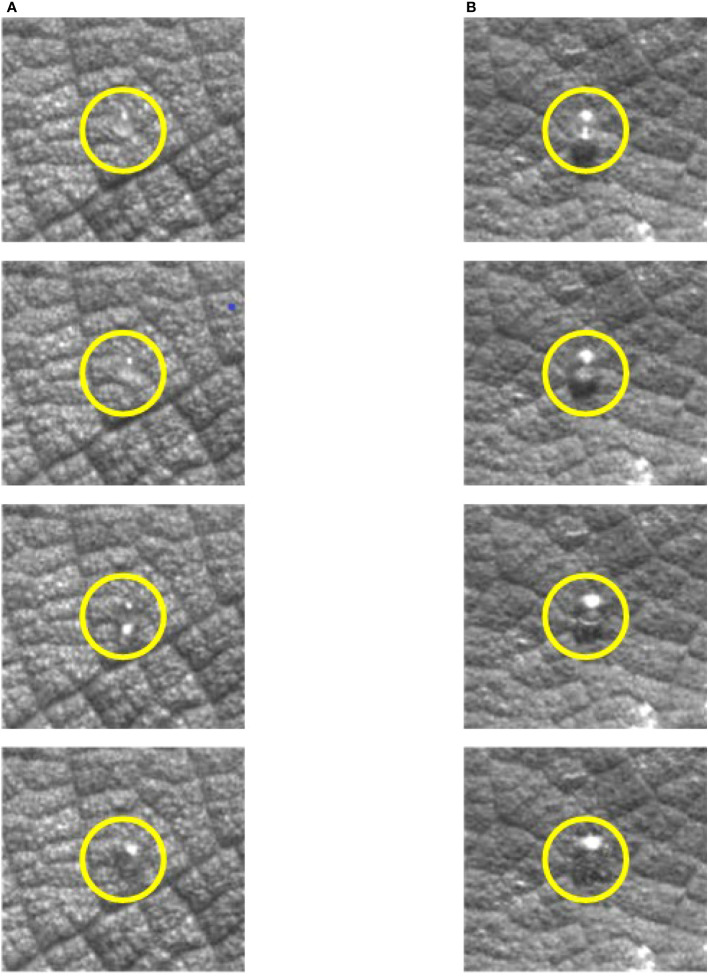
Comparison of droplets of the same mass with and without bubbles. **(A)** Droplets without bubbles. **(B)** Droplets with bubbles.

**Figures 7D-F** show the $\xi_{t}$ of the droplet in the glyphosate solution with 0.25% silicone added. It can be seen in **Figure 7D** that the $\xi_{t}$ of the 1.776 m/s droplet was higher than that of the 1.979 m/s and 2.212 m/s droplet. The spreading factor of the droplet with a velocity of 1.979 m/s is higher than that of the droplet with a velocity of 2.212 m/s. Adding silicone additives to reduce the surface tension of the solution (**Table 3**), the droplets had difficulty maintaining their spherical shape after impacting the blade, which was conducive to spreading. The droplets without bubbles at low velocities had a better spreading factor $\xi_{t}$ . In **Figure 7E**, the relationship of $\xi_{t}$ of the droplets at the three velocities can be seen. Droplets impacting the blade at 1.979 m/s could achieve a good spreading process and the maximum spread area was reached at 1 ms, while droplets with velocities of 1.776 m/s and 2.212 m/s reached the maximum spread area at 1.75 ms and 2.00 ms, respectively. The droplet at a velocity of 1.979 m/s was relatively stable in the oscillation retraction stage, and the variation of $\xi_{t}$ was small. The droplet of 1.979 m/s in **Figure 7F** also had a better spreading effect. Compared with the droplets with the other two velocities, the maximum spread area of the droplets with a velocity of 1.979 m/s was reached at approximately 1 ms, the $\xi_{t}$ was higher, and the effect was better in the spreading process. The 2.212 m/s droplets had a small $\xi_{t}$ value due to the generation of air bubbles. This was because before the droplet impacted the blade, there were obvious bubbles in the droplet, which resulted in no change in the mass of the droplet, but its volume increased (**Figure 8**). The droplets actually hit the blade as a liquid film during impact, and the liquid film breaks up to create a large spreading area. The appearance of air bubbles substantially increased the diameter of the droplet, resulting in a smaller $\xi_{t}$ . Through **Figures 7D–F**, it could be seen that the droplet of 2.212 m/s had a lower $\xi_{t}$ during the spreading process, while the droplets of 1.776 m/s and 1.979 m/s with lower velocities had higher $\xi_{t}$ during spreading.

From **Figures 7G–I**, the spreading factor $\xi_{t}$ of the droplets with the concentration of 0.5% silicone additives was reduced compared with the addition of 0.25% silicone. The number of droplets with bubbles was found to increase significantly after the increase of additive concentration in the experiments. The same phenomenon was found in droplets of three diameters at 0.50% of the additive concentration. The droplets with a velocity of 1.979 m/s had a higher $\xi_{t}$ than the droplets with the other two velocities and quickly reached the maximum spread area. The 1.979 m/s droplets of the three diameters reached the maximum spread area at 1.00 ms, 0.75 ms, and 1.00 ms, respectively. It was roughly 0.25 ms ahead of the droplets with 1.776 m/s and 2.212 m/s velocity. Comparative analysis of **Figures 7G-I** showed that in the three diameters, the spreading process $\xi_{t}$ of the 576-μm-diameter droplet was higher, especially comparing the three diameters with a velocity of 2.212 m/s. The $\xi_{t}$ value of the droplet was a decreasing trend with the elevation of the diameter. The other two velocity droplets had a similar trend but were not as pronounced as the high-velocity droplets. It can be seen from **Figures 7J-L** that only droplets with a diameter of 726 μm reached $\xi_{t}$ above 3 at a velocity of 1.776 m/s.

## 4 Discussion

This study controlled the velocity of droplet impacting the leaf surface by adjusting the height of droplet release. Adding additives of different concentrations changed the surface tension of the droplets. A high-speed photographic system was used to observe the spreading process of droplets. The change of spread factor during the spreading process was analyzed, and the final spread area of different droplets was compared. Four stages of the droplet spreading procedure were observed. A similar phenomenon has been observed in the test on droplet impacting soybean foliage (burr surface) (Jia et al., 2013). During the spreading process, the droplets were spread many times, contracted, and finally stabilized. However, Jia’s study focused on the retracting and broken state after the droplet hit the leaf surface. Studies have shown that the kinetic energy inside the droplets is different when droplets of different viscosities collide (Kumar et al., 2017). The variation of kinetic energy would greatly affect the behavior after the droplet impact, which corresponds to the result of this study. The same height as the released droplets would have the same kinetic energy. Surface tension and viscosity of droplets would vary after the addition of additives, resulting in a completely different impact behavior when droplets hit the blades. The droplets with low surface tension spread the maximum area in this study. It was observed that the liquid spread and flowed from the middle to the surrounding area and formed a circle at the edge of the droplet. This phenomenon indicated that the liquid was less disturbed during flowing. The reason was that droplet surface tension was light, which helped maintain their original spherical shape easier. Thus, it was lightly affected by the burr of the blades. The surface tension during the spreading process was the resistance to the spreading of the droplets. The increasing concentration of silicone additives decreased the surface tension and contributed to lower obstruction of droplets in the spreading process. Different droplets behaved differently at each impact stage. When the droplet spread to the maximum area after impacting the blade, the kinetic energy of the droplet was converted to surface energy. Then, the surface tension of the droplets would cause retraction. Since no rebound occurs, the surface energy of the droplets was converted into viscous dissipation in contact with the leaf surface. More kinetic energy was converted into surface energy. For this reason, the retraction time was faster. The surface tension of droplets was reduced by the addition of silicone additives. The droplets were less hindered by surface tension during the spreading process of the maximum area. Thereby, the spread area was expanded. Thus, there would be more energy consumed by friction with the leaf surface, resulting in a progressively smaller retraction time. Finally, in the balancing process of the droplet, the surface energy of the droplet could be gradually converted into translation kinetic energy and oscillating kinetic energy. There was gradual stabilization of droplets on the leaf surface after gradual energy balance. Glyphosate solution droplets have more surface energy; thus, they required more energy conversion and a longer equilibrium time. This was proved by the equilibration times of the three droplets with the addition of silicone additives, which were 1.25 ms, 1 ms, and 0.5 ms, respectively.

This phenomenon could be attributed to the surface tension and viscosity of the droplets. Some experiments have found that lower surface tension promotes greater diffusion and dampens diffusion oscillations, while higher viscosity inhibits diffusion and retraction processes (Andrade et al., 2012). The spreading of droplets with different surface tensions was indeed observed in the experiment. Droplets with higher surface tension had shorter time to expand to the largest area. The study obtained similar conclusions by analyzing the differences between individual droplets from an energy perspective. By comparing and analyzing the final spread area of various droplets, the droplet spread area of glyphosate solution with additives was much higher than that of droplets of glyphosate solution without additives. Some scholars have found that the addition of additives to pesticides can effectively improve the deposition properties of the liquid on the leaf surface of the target (Song et al., 2021). The same conclusion was found in Song’s study. However, it mainly studied the effects of different kinds of additives on droplet deposition and did not study the effect of additive concentration and the physical properties of droplets on the deposition behavior of liquid droplets.

In this study, it was also found that the increase of additive concentration could increase the final spread area of the droplets. However, the effect of the improvement gradually declined. After comparing the effect of water droplets on glass and leaves with the addition of additives, it was found that the increase in additive concentration could reduce and eliminate the bounce or splash of water droplets, thus improving the diffusion area (Dong et al., 2015). The surfaces used in that experiment were all smooth surfaces, and no more studies were conducted on burrs. The reason for this phenomenon was that increasing the concentration of the additive reduced the surface tension and the contact angle of the droplet (**Tables 2**, **3**), thereby increasing the wettability of the liquid. The effect of surface wettability on droplet kinetics has been studied. It was proposed that droplet diffusion was influenced by the wetting state of the surface (Abubakar et al., 2020). Therefore, increasing the contact angle of the surface would reduce the diffusion diameter of the droplet on the surface. The spreading time of droplets varied with the wetting state of the hydrophobic surface, resulting in the increase in droplet contact angle reducing the spreading time of droplets on the surface. The increase in the static contact angle of the droplet reduces the spreading ability of the droplet on the surface. In this way, droplets can easily form spheres and run off. It was found that increasing the concentration of the additive could lead to the increase of spread area of the droplets. In this study, the correlation between the spread area and the impact velocity of the droplet was found to be significantly positive when the droplet diameter of glyphosate solution was 576 μm. Jia et al. (2013) proposed that the higher the impact velocity of droplets, the greater the maximum spread area. It was similar to the results in this test. It has also been proposed by other scholars that increasing the diameter of the droplet increases the diameter of the wetted area on the impact surface (Abubakar et al., 2021). The same phenomenon was also observed in this test, in which the spread area of the droplet impact burr surface increased with the increase of droplet diameter.

By analyzing the spreading factor of droplets, it was found that the spreading factor of droplets gradually decreased with the increase of additive concentration. It was found that the number of droplets with bubbles increased after the addition of additives. The reason for the phenomenon of decreased spreading factor was the fact that the bubbles were generated from the liquid droplets. When generating droplets, the volume of the droplets increased when air entered the droplets. A bubble would be generated in the droplets, increasing the initial diameter of the droplets $D_{0}$ . It was found that when the concentration of the additive increased, the surface tension of the droplets decreased and the number of droplets producing bubbles became larger, which led to a decrease in the spreading factor. For two droplets of the same mass, the droplet with bubbles could produce a larger spread area when it impacted the blade. This also verified the relationship between the spread area of droplets and the concentration of additives. The hydrodynamics of water droplets on surfaces with a different wettability was investigated, and it was found that droplets with a smaller diameter possess larger spreading factors (Khurana et al., 2019). In our test, it was observed that droplets with a lower velocity had larger diffusion factors. During the test, it was observed that larger droplets could easily form bubbles before impact, increasing the volume of droplets. As the droplet was pushed out of the mouth of the tube, the diameter of the area where the droplet was attached to the syringe gradually decreases. Then, after a small oscillation, the droplet would change to a flattened ellipsoidal shape. In this case, the droplet with a larger mass undergoes a greater shape change than the droplet with a lower mass. The change in droplet shape led to the increase in the contact area of the droplet with air and downward movement by gravity. This led to the increase in the possibility of air entering the droplet. The property of surface tension of such droplet tend to be minimized. The premise of air entering the droplet and forming bubbles was that the pressure generated by the gas inside the droplet and the pressure inside the droplet would be balanced between each other. According to the gas properties, the gas pressure was inversely related to the gas volume for the same mass of gas. Compared to droplets with a larger surface tension, droplets with a lighter surface tension would form larger bubbles inside the droplet.

## 5 Conclusion

In this work, the effect of droplets on the physicochemical properties of the impact burr surface was investigated. By observing the spreading process of droplets, various behaviors of droplets during impact were observed, including movement, spreading, retraction, and balance processes. By analyzing the physical properties, such as the surface tension of droplets and the energy conversion process of droplet diffusion, it was found that higher surface tension inhibits the diffusion process, reduces the retraction time of droplets, and increases the equilibrium time.

Impact velocity of droplet, droplet diameter, and additive concentration will positively affect the final spreading effect of droplets. ANOVA results presented that the additive concentration, droplet impact velocity, and droplet diameter had a significant correlation to the spread area of droplets. Concentration:velocity, concentration:diameter, velocity:diameter, and concentration:velocity:diameter interactions had a significant effect on the spread area of droplets. The spreading effect of droplets with a high concentration and high velocity was found to be better. It was also concluded that the droplets with a higher auxiliary concentration and larger diameter spread better. Velocity was also an important factor, but with a lower effect than the additive concentration and droplet diameter. The curve of surfactants reducing the surface tension of liquids shows a sharp drop and then levels off. This means that when applying pesticides, the addition of more additives can still have a better effect. However, choosing the best concentration in the application process can significantly reduce droplet flow and spray drift, and also reduce the amount of surfactant. This study is relevant in guiding pesticide spraying.

## Data availability statement

The original contributions presented in the study are included in the article/supplementary material. Further inquiries can be directed to the corresponding author.

## Author contributions

PW, CL, LW, and HL contributed to conception and design of the study. PW provided the materials and instruments for the investigation. CX and PW organized the database. PW, CX, and QN performed the statistical analysis. CX wrote the first draft of the manuscript. PW, QN, and HL wrote sections of the manuscript. All authors contributed to manuscript revision, and read and approved the submitted version.
